# Machine Learning in Nursing: A Cross-Disciplinary Review

**DOI:** 10.7759/cureus.87181

**Published:** 2025-07-02

**Authors:** Dimitrios Kosmidis, Dimitrios Simopoulos, Konstantinos Anastasopoulos, Sotiria Koutsouki

**Affiliations:** 1 Department of Nursing, Democritus University of Thrace, Alexandroupolis, GRC; 2 Department of Medicine, Democritus University of Thrace, Alexandroupolis, GRC; 3 Electrical and Computer Engineering, University of Patras, Patras, GRC; 4 Intensive Care Unit, General Hospital of Kavala, Kavala, GRC

**Keywords:** artificial intelligence, clinical decision support, machine learning, nursing, nursing informatics, predictive analytics

## Abstract

Rapid advancement of artificial intelligence (AI) and machine learning (ML) is transforming healthcare, and nursing practice is inevitably affected. Yet limited formal education leaves many nurses hesitant to integrate such tools into everyday care.

This cross‑disciplinary review (i) introduces the fundamental concepts, core types and common evaluation metrices, illustrated with nursing-specific examples; (ii) catalogues the main algorithmic applications in nursing research; and (iii) describes ethical and practical challenges in their clinical use.

A structured search of PubMed, Embase, Scopus, IEEE Xplore and ACM Digital Library (2015-2024) retrieved 1445 records; after de-duplication and screening against inclusion criteria (peer-reviewed, English-language studies that developed, implemented or evaluated an ML method in a clinical, community-health or educational nursing context), 61 papers were analyzed. Supervised approaches predominated, while unsupervised and semi-supervised techniques were less common. Models were evaluated mainly with accuracy, area under the receiver operating characteristic curve (AUROC), precision-recall and F1-score for classification, or mean-absolute/squared error for regression.

Applications spanned eight main categories: (1) predictive risk assessment and early‑warning systems; (2) clinical decision support and diagnostic aid; (3) continuous patient monitoring; (4) workflow, staffing and operational optimizations; (5) documentation and information extraction via natural‑language processing; (6) education and competency development; and (7) other niche applications.

Key barriers to wider adoption remain regulatory and ethical constraints, data quality, model transparency and engagement issues, data challenges, lack of ML-specific training in nursing curricula, and operational limitations. The utilization of ML in nursing is based on three core pillars: strong interdisciplinary collaboration, systematic integration of ML at all levels of nursing education, and a guiding framework that maps human-centered nursing interventions to interpretable ML tools. Such a foundation will enable nurses to safely leverage the results of algorithms, avoid biases and risks, and integrate ethical responsibility into technology-enhanced care.

## Introduction and background

Interdisciplinary collaboration between nursing and informatics can significantly impact patient care [1-3]. Nursing science contributes to extensive clinical experience, established theories, and ethical frameworks, rooted in human interaction - elements challenging to analyze or interpret mathematically [4]. Identifying complex patterns to provide personalized health predictions requires collecting and processing large volumes of heterogeneous clinical data. The technical language of informatics, particularly in machine learning (ML), often refers to advanced algorithmic models as ‘black boxes’ (low interpretability models). This terminology underscores concerns about bias, security, ethical accountability, and the real-world applicability of these systems [5]. However, the profound transformations brought about by artificial intelligence (AI) applications - such as predictive monitoring systems that alert nurses early on to patient deterioration [6] or natural language processing tools that transform clinical documentation [7] - cannot be considered as just another technological innovation. Instead, they require a new understanding and acceptance as an emerging reality that radically affects nursing care.

The applications of AI in nursing can offer significant benefits in various areas of practice. AI technologies can improve clinical decision-making, disease management, patient engagement, support administrative tasks, and transform education and research activities [6,8]. AI applications in nursing can also be used in risk assessment through predictive analytics and natural language processing for faster documentation with greater accuracy [7,9]. Improving efficiency reduces administrative burden and gives nurses the opportunity to focus more on direct patient care [10]. Future research can therefore focus on leveraging AI in multiple nursing fields and roles, as well as linking care to patient outcomes [9]. However, the adoption of AI in nursing remains particularly slow and limited, which is probably related to challenges such as ethical issues, difficulty in acceptance, lack of collaboration, and lack of specialized training [11,12].

Nurses can remain relevant in a technologically advanced future by deciding which aspects of their practice can be entrusted to technology and ensuring the universal elements of human care persist within new systems [13]. This cross-disciplinary review aims to highlight the fundamental concepts and primary categories of ML, describe its applications in nursing practice, and discuss ethical and practical challenges arising from its utilization.

The current review is structured as follows: In the first section, we examine the basic concepts of ML and categorize common techniques, providing simple and understandable examples related to the field of nursing. The next section presents practical applications of ML in nursing, and finally, the last section summarizes the most important challenges and ethical aspects of AI that have been identified in the nursing literature. The review concludes with some general recommendations for integrating AI into nursing education and practice.

## Review

Search methodology

We conducted a structured literature search to identify peer-reviewed articles published between January 1, 2015, and December 31, 2024, focusing on the application of AI and ML in nursing-related contexts. The search was performed across five electronic databases: PubMed, Embase (via Summon), Scopus, IEEE Xplore, and the ACM Digital Library. For each database, we constructed tailored search strategies combining three key concepts: (A) ML technologies (e.g., “machine learning,” “deep learning,” “neural networks”), (B) healthcare or clinical application, and (C) nursing involvement (e.g., “nursing informatics,” “registered nurse*,” “nurs*”).

We included papers that satisfied all three conditions below: (a) ML term: the study designs, implements, or evaluates at least one ML method; (b) healthcare context: the work is conducted, tested, or explicitly aimed at a clinical, community-health, or educational healthcare setting; (c) nursing relevance: the application targets nursing care, education, or management, and nurses are identified as key users/beneficiaries of the system.

We excluded papers that (a) relied solely on rule-based expert systems or conventional statistical models with no learning component; (b) focused on general fitness or health-tracking apps, animal-health work, or computer-only laboratory studies that lacked a clearly stated clinical application; (c) omit the architecture, the algorithm’s name, the training process, dataset, and other technical details, thereby preventing meaningful analysis of their ML modalities; and (d) lacked a peer-reviewed, full text in English, was a review, an editorial, commentary, protocol, or conference abstract without a full paper, or fell outside the time frame. The search strategy is reported in accordance with the Preferred Reporting Items for Systematic Reviews and Meta-Analyses (PRISMA) checklist and the full search strategies, and database-specific details are provided in the Appendix.

**Figure 1:**
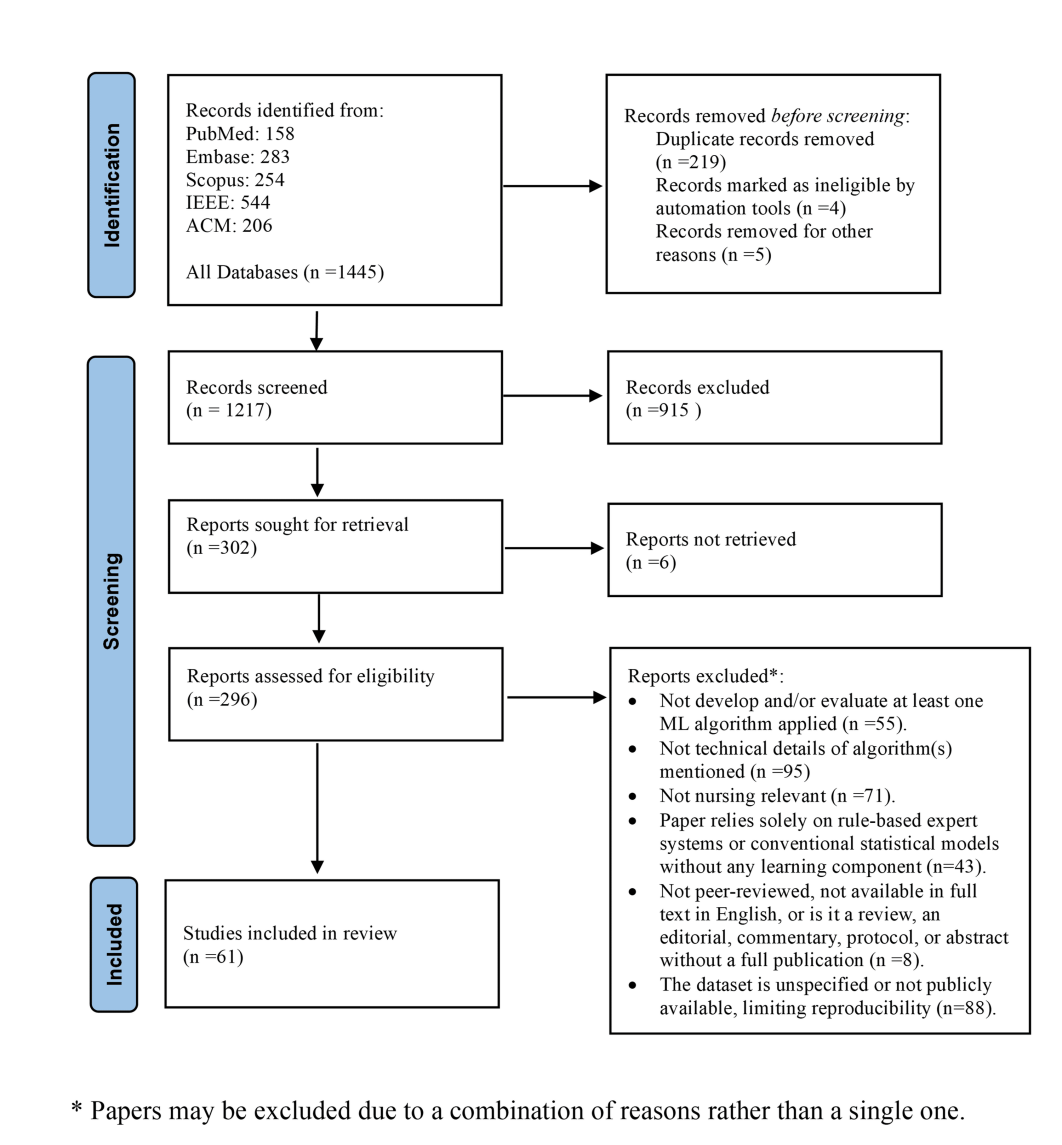
Preferred Reporting Items for Systematic Reviews and Meta-Analyses (PRISMA) 2020 flow diagram ML: machine learning

Two of the authors (DK, DS) independently screened all titles, abstracts, and full texts against the inclusion-exclusion criteria; any disagreements were resolved by a third reviewer (GA). To ensure balanced disciplinary coverage, we followed a bidirectional process: ML-focused papers were first appraised by informatics experts and then by nursing reviewers, while nursing-grounded studies followed the reverse order. Each included article was subsequently evaluated with a five-item checklist proposed by von Gerich et al. (clarity of aim, dataset/algorithm transparency, clinical-nursing relevance, reproducibility, ethical reporting) [14]. The complete screening flow is shown in the PRISMA diagram (Figure 1). During data extraction we also recorded whether each study explicitly addressed nursing-specific benefits or challenges and consulted key methodological sources to frame ML concepts and evaluation metrics.

Results

From 1445 records identified across five databases, 61 eligible studies remained for detailed synthesis. The results are presented in three parts, moving from foundational ML concepts to nursing-specific applications and, finally, to the ethical and practical challenges they entail.

Fundamental Concepts of Machine Learning

Haenlein et al. [15] in their article "A Brief History of Artificial Intelligence: On the Past, Present, and Future of Artificial Intelligence", define AI as “the capability of a system to correctly interpret external data, learn from these data, and utilize this learning to accomplish specific goals and tasks through adaptive adjustments”. ML specifically refers to the ability of systems learning from training data related to problems, thereby automating the generation of analytical models and solving relevant tasks [16]. According to Janiesch et al. [16], ML is considered a subset of AI, focusing on enabling computers to learn from data and enhance their performance on tasks without explicit programming. It involves using algorithms to automatically build models capable of making predictions or decisions based on input data.

ML is rooted in the concept of extracting knowledge directly from data itself rather than explicitly applying pre-defined rules. It utilizes algorithms designed to recognize patterns, correlations, or relationships within these data and subsequently suggests autonomous decisions based on these patterns [16-18]. For example, consider a dataset comprising patients' vital signs, such as body temperature, heart rate, and blood pressure. Without explicitly defining what is the "normal" or "abnormal," an algorithm identifies that most observations exhibit body temperatures ranging between 36.5-37.5°C, while certain values significantly deviate (e.g., temperatures around 39°C). Without prior knowledge of what constitutes normal, the algorithm autonomously "learns" from the data that these deviations likely represent abnormalities. Subsequently, observations with temperatures ≥39°C, combined with elevated heart rates and blood pressure, may be classified as indicative of a pathological condition, such as an infection. Thus, learning originates directly from the data, even without previous knowledge, making the algorithm’s decision autonomous.

Algorithms constitute finite sequences of instructions executed within a finite period to solve computational problems and extract inferences from data [19]. In the context of ML, a model typically incorporates at least one or more algorithms. These models are trained and applied to datasets aiming at predicting or classifying outcomes by identifying underlying patterns. After training, the model "learns" from the existing data and applies the gained knowledge to predict or classify new, unseen information/data. Indicative examples of widely recognized ML algorithms include Decision Trees, K-Nearest Neighbors (KNN), and Neural Networks (NN). Decision Trees is a well-known ML algorithm, which makes predictions based on recursive data partitions, leading to interpretable results. KNN guesses by copying what the closest examples of data do. A neural network functions as a multi-regulator of data: each data connection is adjusted according to the data it sees. Thus, the overall result is closer to the correct answer. If these connections and micro-adjustments are stacked in multi-level layers, it is possible to extract complex patterns, providing solutions to very complex problems [20]. The aforementioned algorithms are further explained in the following sections.

Types of Machine Learning and Healthcare Applications

**Figure 2:**
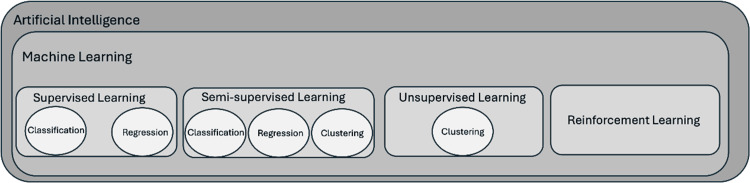
Brief hierarchical overview of artificial intelligence (AI): Indicative machine learning (ML) types and their core tasks.

ML and its models include several learning types, each suitable for different analyses and data structures. ML models follow various basic techniques classified into four main categories: supervised learning, unsupervised learning, semi-supervised learning, and reinforcement learning. Figure 2 depicts the discrete ML types and their main tasks.

Supervised Learning

In supervised learning, models are trained using labeled data, involving two primary methods: classification and regression. Classification is commonly used for categorical data. "Labels" in ML datasets refer to the information or categories associated with each data sample, identifying the desired outcome or group to which the sample belongs. Essentially, labels represent the correct answers or targets that a model is expected to predict. For example, if the model must classify fruit images as apples or oranges, the labels would be "apple" and "orange." Labels are crucial in supervised learning because the model is trained based on input data and their corresponding outputs. Once the model learns the relationship between inputs and outputs, it can categorize new input data effectively [21].

Regression analysis is a statistical method used to model how a specific dependent outcome - such as the length of a patient’s hospital stay - is related to one or more independent variables (predictors). By examining data like patient age, laboratory values, and comorbid conditions, regression quantifies the strength and direction of these relationships [21,22]. In ML, regression models predict continuous outcomes; for example, Li et al. [23] built a regression-based ML model to estimate the hospital‐stay duration of HIV patients using demographic information, clinical measurements, and comorbidity data as input features. Another example involves predicting systolic and diastolic blood pressure based on specific clinical features. Using data like age, weight, exercise level, and electrocardiograms, the ML model is trained to identify relationships between these characteristics and actual blood pressure values. The data inputs are referred to as features, while the systolic and diastolic blood pressure values, which the model learns to predict, serve as labels [24].

Common supervised learning algorithms used in healthcare include Support Vector Machines (SVM), which help categorize data by finding optimal separation among categories, and Naïve Bayes, a simple, fast algorithm particularly effective with large datasets. Both methods can likewise be adapted for semi-supervised learning when only part of the data is labeled [25,26]. Decision Trees are popular algorithms due to their simplicity, clarity, and explainability, commonly used in predicting conditions such as cardiovascular diseases. The Random Forest (RF) algorithm integrates multiple decision trees to yield more accurate predictions and often outperforms other algorithmic methods [26,27]. The KNN algorithm classifies data based on proximity (assuming data points near each other likely belong to the same category) but has been criticized for poor performance with high-dimensional data (data with very many variables compared with the number of samples) and a high risk of overfitting (the model memorizes the training data and fails to generalize to new data). Artificial Neural Networks (ANNs) mimic the human brain to find patterns and relationships in complex data, making them particularly useful for tasks such as medical image or signal analysis [25,28].

Logistic regression, although a traditional statistical method, is typically implemented and viewed as a classification algorithm in the context of ML, especially for binary classification problems. Due to these properties, logistic regression has been applied effectively in COVID-19 patient triage, accurately predicting patient severity in emergency departments [29]. A relatively new family of algorithms, Gradient Boosting, including variations such as XGBoost, LightGBM, and CatBoost, utilize advanced techniques and are particularly valuable in clinical research due to their high accuracy and fast training capabilities [30]. Each of these algorithms possesses unique advantages, making them suitable for different scenarios [31]. In nursing, examples of studies employing supervised ML include predicting patient readmissions [32], classifying nursing notes or observations [33], and predicting patient pain scales among others [34].

Unsupervised Learning

Unsupervised learning is a different approach within ML that operates without the use of labels, meaning there is no predefined prediction of specific outcomes [35]. In unsupervised learning, models analyze data primarily through techniques like clustering and association analysis [21].

Clustering identifies patterns within data, such as categorizing patients with similar symptoms or genetic profiles based on their similarities. It operates on the logic that data within the same cluster are more like each other, whereas data from different clusters exhibit greater differences. For example, clustering can categorize patients based on glucose levels or blood pressure readings, forming risk groups aimed at disease prevention or treatment. Additionally, clustering can detect abnormalities such as tumors or lesions from MRI or other imaging data and categorize diseases or predict progression in chronic conditions [21,36]. Nurses, for instance, could use clustering to categorize infection patients based on shared characteristics (infection type, symptoms, test results, medical history). Classifying infections as mild, moderate, or severe can aid various nursing decisions, such as personalized care plans, preventive measures, and improved antibiotic management.

Association analysis operates on the logic that "if A occurs, B is likely to occur" and extracts association rules accordingly. The Apriori algorithm is among the most recognized association analysis methods [37]. For example, to determine side effects commonly appearing together in patients taking multiple medications, the algorithm initially identifies frequent individual side effects (e.g., dizziness, nausea, hypoglycemia, fever). These are then combined into frequent pairs, rare combinations are discarded, and the process iterates to identify additional frequent side effects. This approach enables nurses to adjust medication management by anticipating potential symptoms. Association analysis significantly contributes to discovering relationships between diseases or treatments [19]. For instance, Lakshmanarao et al. [38] identified features such as age, cholesterol, and maximum heart rate as having increased correlations and probabilities of heart disease occurrence.

Dimensionality reduction techniques are often considered unsupervised learning, as their primary goal is understanding data structure without needing labels. They simplify complex data with numerous features (or dimensions), highlighting the most significant elements for analysis [39]. Genetic data, for example, can have thousands or millions of features (genes, mutations, DNA sequences), complicating full-scale analysis [40]. Techniques like Principal Component Analysis (PCA), Kernel PCA (KPCA), and t-Distributed Stochastic Neighbor Embedding (t-SNE) allow transforming complex data into smaller feature sets without losing essential information. PCA finds new combined features that capture most of the differences in the data, so the dataset can be represented with fewer variables; KPCA extends PCA by mapping data into a higher-dimensional space before reduction, enabling the capture of nonlinear patterns; and t-SNE preserves local relationships to project high-dimensional data into two or three dimensions for visualization. These methods help reveal the factors most relevant to diagnosis or treatment effectiveness [41].

Unsupervised learning, not requiring predefined categories, can significantly enhance understanding hidden patterns in medical data, opening new avenues for analysis and information extraction in healthcare [21]. Among these approaches, Data Envelopment Analysis (DEA) is also an unsupervised method that has been used in nursing research. Cosgun et al., for example, applied DEA combined with ML techniques to evaluate nursing home efficiency during the pandemic [42].

Semi-supervised Learning

Semi-supervised learning combines supervised and unsupervised learning techniques to improve the accuracy of model training. Specifically, semi-supervised learning uses a small number of labeled data along with a large set of unlabeled data. This approach is particularly useful when data labeling is costly or time-consuming, and a substantial volume of unlabeled data is available [21]. For instance, consider developing a model predicting patient risk based on blood pressure and body temperature. The available labeled data from a small patient group might identify blood pressure ≥140/90 and temperature ≥38.5°C as "at risk," while blood pressure ≤120/80 and temperature ≤36.8°C are labeled as "no risk." Simultaneously, there exists a large unlabeled patient dataset with various blood pressure and temperature readings. In semi-supervised learning, the model initially learns from the labeled "at risk" and "no risk" data, correlating blood pressure and temperature to patient conditions. The algorithm then applies this knowledge to unlabeled data, identifying risk patterns such as elevated pressure and temperature. Hence, the model predicts that a patient with a blood pressure of 145/92 and a temperature of 38.8°C is "at risk," whereas another patient with blood pressure 125/80 and a temperature of 36.5°C is "no risk." In the real world, however, clinical risk-assessment models incorporate multiple patient characteristics - such as laboratory results, comorbidities, and demographic factors - to improve predictive accuracy and better capture patient complexity.

Semi-supervised learning algorithms are widely applied in medical image analysis. Additionally, algorithms like SVMs are frequently used in classification problems, such as Alzheimer's disease detection. While these algorithms remain less prevalent than supervised learning algorithms, they are increasingly popular as alternatives in scenarios with limited labeled data [43]. In nursing, an example combining supervised and semi-supervised learning is the study by Wen et al. [44]. A small set of labeled physiological signals (heart rate, respiratory rate) from remote health-monitoring devices was used to guide initial training, and a larger pool of unlabeled signals then refined the model. By first training on known “normal” versus “abnormal” signal patterns (supervised step) and subsequently allowing the model to iteratively assign pseudo-labels to unlabeled data (semi-supervised refinement), the authors improved prediction of physiological deterioration in home-based elderly care [44].

Reinforcement Learning

Reinforcement learning (RL) is a computational approach where algorithms learn through trial and error. Decisions are made in sequences of steps, each potentially yielding positive or negative outcomes. The overall result aggregates these outcomes, and the algorithm's goal is to identify the optimal strategy that maximizes positive outcomes or minimizes negative ones [45]. Reinforcement learning algorithms can be likened to a person learning to play a game - receiving rewards for correct actions and penalties for mistakes, thus learning through continuous reinforcement which actions lead to the best results [46,47]. Although sometimes compared with semi-supervised approaches due to the use of unlabeled data in some settings, reinforcement learning is generally recognized as a distinct paradigm in which an agent learns by interacting with an environment and receiving rewards. On rare occasions, researchers combine reinforcement learning with unsupervised or semi-supervised techniques - i.e. using RL to refine representations learned from unlabeled data [45].

An example in healthcare could be a system managing medication dosage for a hypertensive patient. The system learns through trial and error, analyzing blood pressure data before and after medication administration, identifying potential side effects, and adjusting dosages accordingly. Initial data input involves an initial medication dosage evaluated for effectiveness. If blood pressure normalizes without side effects, the system receives a reward; if the pressure remains high or side effects occur, it receives a penalty. With continuous data input, the system learns optimal dosage adjustments, ultimately recommending the most effective dosage.

Applications for reinforcement learning in healthcare include Dynamic Treatment Regimes, where therapeutic strategies adapt dynamically to patient conditions, providing personalized medication for chronic diseases such as cancer, diabetes, anemia, and HIV [45,47,48]. Additional studies apply reinforcement learning in intensive care units to optimize clinical decision-making (e.g., sepsis treatment, sedation management, mechanical ventilation) and to support hospital operations (e.g., operating room scheduling). Furthermore, reinforcement learning can support disease prevention and community health management (e.g., predicting outbreak hotspots, prioritizing vaccination campaigns, and optimizing resource allocation), thereby enhancing public health outcomes and efficient use of resources [45].

Despite its growing prominence in personalized medicine and robotics, reinforcement learning remains less prevalent overall in healthcare compared to supervised learning, which is currently the dominant approach [47,49].

Evaluation of ML Algorithms

ML does not produce perfect results; therefore, ML models and algorithms must undergo evaluation. The evaluation of ML algorithms relies on various metrics to assess their performance, aiding researchers and practitioners in determining the effectiveness of ML models. Proper metric selection depends on the problem type (classification, regression, clustering), aiming for optimal functionality and result interpretation [50].

Common classification algorithm metrics include accuracy, precision, recall/sensitivity, specificity, and F1-score [51]. Accuracy is the proportion of correct predictions (both positive and negative) out of all predictions made. Analogously, precision represents the proportion of true positive predictions out of all positive predictions made. Recall or sensitivity is the proportion of true positive predictions out of all actual positive cases present in the data. Similarly, specificity expresses the proportion of true negative predictions out of all actual negative cases present in the data. Finally, the harmonic mean of precision and recall/sensitivity defines the F-score balancing precision and recall into a single value - especially important when dealing with imbalanced datasets [51].

Another well-known graphical evaluation method is the Area Under the Curve (AUC) of the Receiver Operating Characteristic (ROC), evaluating an algorithm’s capability to distinguish between categories across various thresholds, providing an overall performance picture. Additional metrics include the Matthews Correlation Coefficient (MCC) [52], the Precision-Recall Curve - particularly useful for imbalanced datasets - and Balanced Accuracy Rate (BCR), which balances sensitivity and specificity [53]. 

For regression problems, metrics typically measure the deviation of predictions from actual values. Although no consensus on a single "best" metric exists, commonly used metrics over the past 25 years include Mean Squared Error (MSE), Root Mean Squared Error (RMSE), Mean Absolute Error (MAE), and Mean Absolute Percentage Error (MAPE) [48]. The R coefficient (Pearson correlation coefficient) and R² (coefficient of determination) evaluate how well a statistical model explains variance in the dependent variable [51]. However, a few researchers criticized the metrics above as biased, inadequate, or misleading for evaluating predictive model accuracy for numerical data [54]. 

For clustering algorithms, recommended metrics include the Silhouette coefficient, Davies-Bouldin index, and Calinski-Harabasz index [51]. Although these metrics attempt to summarize algorithm performance into a single numeric measure, they often fail to fully capture multidimensional capabilities. To address this, frameworks like "Camilla" by Liu et al. propose using a central multidimensional Model Competence Index. This framework is “a comprehensive attempt for measuring the multifaceted strength of each machine learning algorithm through exploring the connections between the researches on psychometric theories and machine learning evaluation” [55].

The aforementioned metrics are vital to evaluate each respective model, depending on the prediction problem they aim to solve. For instance, a mortality classification model with an accuracy of 98% indicates a high-reliability prediction advising the clinicians in their daily decision-making processes [56]. Similarly, nurses may employ MAE to evaluate the patients’ length of stay accuracy. For example, a MAE value of 1 could indicate a deviation of ± 1 day in a hospital unit.

Beyond classical accuracy metrics, ML algorithm evaluation in healthcare should also consider factors such as inference time (the time required for the computer to complete prediction results), computational resource efficiency, and noise robustness (e.g., incorrect pressure values due to patient movement). These parameters are critical under real-world conditions, and various evaluation approaches have been proposed in the literature. However, no universal evaluation metric exists, as selection depends on the specific problem, analysis objectives, and application field requirements [57].

Nursing Applications

**Figure 3:**
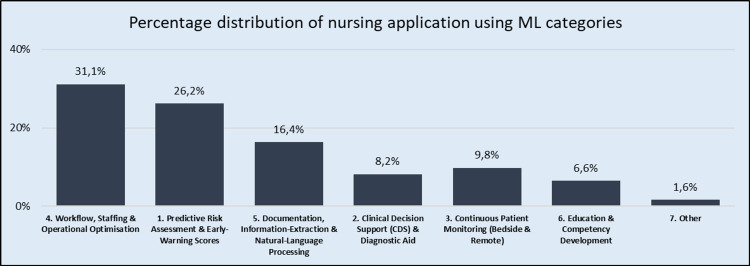
Main categories of machine learning (ML) applications in nursing.

Our literature search yielded a variety of results, which were classified into seven categories of ML applications (Figure 3).

Workflow, staffing, and nurse-function optimization formed the largest paper category, with objectives ranging from forecasting staffing needs and balancing workload to optimizing bed management or predicting supply needs. For example, Alzu’bi et al. [58] adopted a supervised classification strategy - ultimately favoring an artificial neural network trained on 20 demographic, health and workplace attributes from 191 nurses - to label each nurse as “absent” or “non-absent” for the coming year. The resulting model with 82% accuracy, equips hospital managers to flag staff at high risk of absenteeism and intervene early to maintain service quality and productivity. Inoue [59] used RF algorithms to achieve 73.7% accuracy in predicting the next day's workload, which significantly helps in timely shift coverage. Taking a different approach, Nagayoshi and Tamaki [60] introduced reinforcement learning for online schedule revision when sudden nurse absences occur, offering flexibility beyond classical algorithmic schedulers. Hiremath et al. [61] developed a method for automating medication administration by updating inventory and thus saving staff time. In another attempt to predict annual turnover, Kim et al. [62] achieved a very high AUC and accuracy (0.97 and 98.9%, respectively) using an RF model.

The second category concerned predictive risk assessment/early warning scores for the prevention and timely treatment of various dangerous situations involving patients. The most common goals were to identify patients who might experience deterioration or complications so that nurses could intervene earlier. The data used in these cases is extracted from the continuous flow of vital signs, laboratory test results, nurse assessments, and history from electronic health records. In this category prediction risk of pressure injury [63] patients fall [64,65], mortality [56], infection [66], sepsis [67,68], as well as broader monitoring of vital signs to predict various other conditions [69] are some common examples. 

Another application field is documentation, information-extraction and natural-language processing (NLP), used to convert unstructured data - such as free-text nursing notes - into formats suitable for ML analysis [70,71]. In some cases, formulating nursing diagnoses and care plans, such as estimating the risk of patient readmissions based on nurse staffing and experience, represents another promising area [72].

Clinical decision support (CDS) and diagnostic aid is a special field category in which the goal is to provide nurses who are at the patient's bedside or triage nurses with recommendations generated by algorithms, e.g., possible diagnoses, warnings about drug interactions, suggestions for best practices. One such example of an algorithm that classifies patients into levels of agitation-sedation has been developed by Dai et al. [73] to assist ICU nurses by generating recommendations for adjusting the relevant medication. Kajanan et al. [74] compared 10 supervised algorithms to identify the type of respiratory failure based on a dataset of 700 arterial blood gas (ABG) test results. This effort used as a diagnostic aid for doctors and nurses in a public hospital in Sri Lanka and concluded in an XGBoost algorithm yielded the highest average performance (accuracy 98,65 %, MSE 1,35 %). In a different approach, Kaduwela et al. [75] developed nurse-friendly data annotation software based on converting video to frames and automatically placing landmarks on the faces of newborns. After training, the ML model achieved 98% accuracy and 97.7% precision in detecting neonatal pain.

Continuous patient monitoring is another category of applications that is evolving in the field of nursing. Batool et al. [76] developed SecFePAS, an application that derives pain ratings from facial-expression video streams while preserving privacy by encrypting the data in real time. Sheng et al. [77] applied an unsupervised clustering pipeline. First with a principal-component analysis condensed five weekly metrics (blood pressure, weight, physical activity, sleep, self-report logs) from 60 older adults’ data with multimorbidity. Then using k-means algorithms, a supervised clustering approach, grouped participants into three distinct engagement profiles -“least”, “typical”, and “highly” -engaged users. Analysis of the clusters showed that the highly engaged cluster exhibited steadier blood pressure and higher activity levels, highlighting how unsupervised ML can uncover actionable patterns in long-term digital-health use. Smart ML analyses of pressure layers classify various patient positions in bed with 99.6% accuracy and highlight the limbs at risk of pressure ulcers [78]. Patient monitoring is now being extended to the home, such as the deep-CNN platform developed to recognize daily activities from home acoustics to remotely monitor vulnerable elderly people [79].

For training and skills development, Bodur et al. [80] used tree-based ML to show how virtual reality simulation enhances the self-directed learning skills of nursing students, while Adile et al. [81] proposed a deep learning-assisted statistical model that adapts safety training scenarios for healthcare workers in resource-constrained environments. In other cases, the focus is on tools that improve the well-being and ergonomics of nurses. Bangani et al. [82] employed a supervised classification approach - a decision tree algorithm - on ECG data collected with a wearable patch to label each signal window as “stress” or “no-stress.” Their goal was to provide personalized, real-time monitoring of nurses’ stress levels during their shifts. Similarly, Judge et al. [83], in order to prevent musculoskeletal injuries, created a computer vision system that predicts the weight of an object and the perceived strain from lifting based on high-speed skeleton data. These examples complement a wide range of applications demonstrating that the use of ML in nursing and the benefits for patients and nursing care appear to be limitless. Table 1 indicates various ML types providing prediction categories, and their key algorithms, accompanied by specific nursing application examples.

**Table 1:** Machine learning (ML) types, prediction categories, key algorithms, and specific nursing applications examples. PCA: principal component analysis, KPCA: kernel PCA, ABI: abnormal breathing index

ML Type	Algorithm prediction categories	Key Algorithms	Nursing applications
Supervised Learning	Classification	Random Forests	Jahandideh et al. [84] Determine fall-associated factors and develop high-performance prediction tools for fall risk patients in acute and sub-acute care services in Australia
Unsupervised Learning	Clustering	K-Means Clustering	Sheng et al. [77] A mixed (PCA and clustering) analysis were used to group 60 older adults based on their engagement levels, while using digital health platforms.
Supervised & Unsupervised Learning	Regression	PCA, KPCA	Pan et al. [85] Develop an effective fall risk prediction model for community older adults by integrating PCA with machine learning
Semi-Supervised Learning	Classification/ Regression	Support Vector Machines (SVMs)	Yokota et al. [64] Constructed a model using a support vector machine to determine whether an inpatient will suffer a fall on a given day, depending on patient status on the previous day.
Semi-Supervised Learning	Clustering / Classification	Hierarchical clustering / Random Forests	Gupta et al. [86] A semi-supervised approach that clustered unlabeled hydraulic bed-sensor breathing data into six pattern groups is used. Then they trained a classifier to derive a daily index (ABI) for older adults living at home.
Reinforcement learning		Q-learning	Nagayoshi & Tamaki [60] Method for work revision that utilizes reinforcement learning to improve a constructive nurse scheduling system.

Challenges of ML Implementation in Nursing

Ethical concerns associated with AI in general and ML in particular are among the most widely cited issues in literature. These concerns take several forms. One prominent example relates to compliance with data protection regulations such as the General Data Protection Regulation (GDPR). Failure to adhere can significantly compromise patient privacy [87]. Another ethical issue involves the possibility of model failure or error, potentially leading to incorrect healthcare decisions and unclear accountability [14,88]. Ethical challenges also include disparities in access to technology among vulnerable populations or in areas lacking digital infrastructure, as well as bias stemming from reliance on readily available datasets [88,89]. Data security is particularly critical, as there have been documented deaths linked to cyberattacks on medical systems [90]. Adelson et al. [91] highlight these risks, especially the danger of misdiagnosis resulting from AI tools. Taylor et al. [92] identify additional concerns, such as disease-related stigmatization and increased healthcare costs for patients flagged preemptively.

Acceptance of AI and ML tools by nursing staff is another major challenge identified by many authors. These tools are often described as "black boxes." The lack of transparency in algorithmic calculations, combined with limited involvement of nurses in the development and testing phases and insufficient training, can undermine confidence in the accuracy and reliability of ML outputs. As a result, nurses may either reject these tools or struggle to integrate them into practice [14,87,89,93,94]. Von Gerich et al. [14] in their review point to insufficient collaboration between nurses and informatics experts throughout the ML development process. They also identify a lack of accepted reporting guidelines for ML in nursing research and emphasize the need to incorporate basic nursing informatics into education. However, it is worth noting that their review excluded recent conference proceedings, potentially omitting contemporary developments.

Another key issue is the availability of large and high-quality datasets necessary for ML training. Algorithms typically require hundreds or thousands of records for effective prediction. In some domains, such as fall prediction, data volume remains limited, resulting in insufficient training datasets. Imbalanced datasets (e.g., more survivors than non-survivors) may also introduce bias [94,95]. The lack of structured data - especially in nursing, where much data comes from electronic health records (EHRs) - can further restrict model accuracy and reliability. ML models are often developed under controlled conditions, which may not generalize well to real-world clinical settings. As such, deploying these tools in existing hospital systems can be both difficult and time-consuming [14,87,95].

Moreover, many healthcare organizations may lack the resources or strategic infrastructure to support ML deployment. Current systems may not provide the required interoperability, and the development, installation, and maintenance of ML systems can be expensive. Along with the ongoing need for updates, maintenance, and user training, these challenges can hinder integration into daily nursing practice [90-92,95].

## Conclusions

This cross-disciplinary review aims to clarify the basic concepts and categories of ML, providing simple examples relevant to nursing. It presents the introductory steps of ML applications in nursing practice, as well as the implementation challenges arising, such as trust, ethical responsibility and technical difficulties. To realize the added value of ML in clinical practice, both interdisciplinary collaboration and the integration of fundamental informatics knowledge into undergraduate and postgraduate nursing education are essential. 

In addition, the establishment of a framework correlating nursing interventions with ML tools is required. These tools should incorporate or interpret fundamental nursing theories, such as cross-cultural or person-centered care, transforming algorithmic recommendations into nursing practice. Such an approach has the potential to transform nursing care, enabling safer, more efficient healthcare systems, without undermining its ethical and humanistic foundations.

Nurses must be able to accurately interpret and safely apply the results of AI. In the coming years, the engagement of nursing in the complex world of information technology is expected to be considerable. For this to happen, a fundamental knowledge of AI is essential, including an understanding of the basic types of algorithms, their functions, and basic measures of their evaluation. Along with challenges, particularly regarding ethics, imbalanced patient subsets, and data privacy issues, this core knowledge is essential to ensure responsible integration of AI technologies into nursing practice.

## Appendices

**Table 2:** Search Strategies by database, boolean/keywords/filters used, and records retrieved. The search was performed on 2025, February 12.

Database - Platform	Search Fields Used	Search Strategy (Boolean/keywords)	Filters Used	Records Retrieved
PubMed /MEDLINE	MeSH, Title/Abstract ([tiab])LINE	("Artificial Intelligence"[Mesh] OR "artificial intelligence"[tiab] OR "Machine Learning"[Mesh:noexp] OR "Machine Learning"[tiab] OR "deep learning"[tiab] OR "neural network*"[tiab] OR "algorithm*"[tiab] AND ("health care"[tiab] OR healthcare[tiab]) AND ("Nursing"[Mesh] OR "Nursing Informatics"[Mesh] OR nurs*[tiab] OR "registered nurse*"[tiab]) AND ("2015/01/01"[PDAT] : "2024/12/31"[PDAT]) LANGUAGE: English	English; 2015–2024	158
Embase (Elsevier) – accessed via Summon discovery layer, HINARI	EMTREE (exploded), Title/Abstract	"artificial Intelligence" OR "AI" OR "machine learning" OR "deep learning" OR "neural network*" OR "algorithm*"	English; 2015–2024	283
SCOPUS	Title, Title/Abstract/Keywords	TITLE( "artificial intelligence" OR "machine learning" OR "deep learning" OR "neural network*" OR "algorithm*" OR "AI" ) AND TITLE-ABS-KEY ( "health care" OR "healthcare" ) AND TITLE( nurs* OR "nursing informatics" OR "registered nurse*" ) AND ( PUBYEAR > 2014 AND PUBYEAR < 2025 ) AND ( LIMIT-TO ( LANGUAGE,"English" ) )	English; 2015–2024	254
IEEE Xplore	All Metadata Fields	((("artificial intelligence" OR "AI" OR "machine learning" OR "deep learning" OR "neural network*" OR "algorithm*") AND ("health care" OR healthcare) AND (nurs* OR "nursing informatics" OR "registered nurse*")))	English; 2015–2024; Journals/Conferences	544
ACM (The ACM Full-text collection)	All Fields; Abstract; Title	[[All: "artificial intelligence"] OR [All: ai] OR [All: "machine learning"] OR [All: "deep learning"] OR [All: "neural network*"] OR [All: algorithm*]] AND [[All: "health care"] OR [All: healthcare]] AND [[Abstract: nurs*] OR [Abstract: "nursing informatics"] OR [Abstract: "registered nurse*"]] AND [E-Publication Date: (01/01/2015 TO 12/31/2024)]	English (manually); 2015–2024	206
